# Efficacy and cost-effectiveness of extending risk-stratified colorectal cancer screening: evidence from China’s first province-wide program

**DOI:** 10.1097/JS9.0000000000004654

**Published:** 2026-01-12

**Authors:** Juan Zhu, Bingjie Jiang, Chen Zhu, Ruyi Xia, Lili Song, Weimiao Wu, Yi Lou, Lingbin Du

**Affiliations:** a Department of Cancer Prevention, Zhejiang Cancer Hospital, Hangzhou Institute of Medicine (HIM), Chinese Academy of Sciences, Hangzhou, China; b School of Public Health, Nanjing Medical University, Nanjing, China; c Department of Epidemiology and Biostatistics, School of Public Health, Xi’an Jiaotong University Health Science Center, Xi’an, China; d Department of Research, Zhejiang Cancer Hospital, Hangzhou Institute of Medicine (HIM), Chinese Academy of Sciences, Hangzhou, China.

**Keywords:** colorectal cancer, cost-effectiveness, fecal immunochemical test, risk-based screening, screening

## Abstract

**Background:**

China accounts for nearly 30% of global colorectal cancer (CRC) cases but has only 3% screening coverage, underscoring the urgent need to expand coverage. We evaluated the efficacy and cost-effectiveness of extending risk-stratified CRC screening based on the first province-wide, full-coverage initiative in China (2020–2024).

**Methods:**

This population-based study covered 17 780 462 average-risk residents aged 50–74. Those participants were stratified using the Revised Asia-Pacific Colorectal Screening Score (APCS) and fecal immunochemical testing (FIT). High-risk participants (APCS ≥5 or FIT-positive) were referred for colonoscopy. A health care system perspective Markov model simulated a cohort of 100 000 individuals to compare screening strategies by initiation age and frequency. Outcomes included screening yield (participation, detection, and death averted), resource utilization (colonoscopies, costs per lesion detected, and number needed to screen), and cost-effectiveness. Incremental cost-effectiveness ratios (ICERs), benchmarked against three times per capita GDP (USD*$*53 235 in 2023) per quality-adjusted life-year (QALY) gained. Sensitivity analyses were performed.

**Results:**

Among 10 364 955 eligible participants, 1 582 606 (15.27%) were high-risk, with 38.92% colonoscopy compliance. Screening detected 59 954 advanced adenomas (AAs) and 5708 CRCs, with 11 and 108 colonoscopies needed per lesion, respectively. The cost per AA and CRC detected was *$*1588 and *$*16 684. All screening strategies were cost-effective regardless of the starting age or frequency, yielding ICERs of *$*6718–*$*21 177 per QALY. Annual screening starting at 40–44 years achieved optimal effectiveness (19 955 QALYs gained, 1126 CRC deaths averted per 100 000).

**Conclusion:**

This first province-wide, full-coverage screening initiative demonstrates the effectiveness and cost-effectiveness of extending risk-based CRC screening in resource-rich regions of China, supporting expanded coverage and informing policy decisions for CRC prevention in similar settings.

## Introduction

Colorectal cancer (CRC) is a major global health challenge, with 1 926 118 new cases and 903 859 deaths in 2022^[1]^. Notably, China bears nearly 30% of global CRC cases, recording 517 100 new cases and 240 000 deaths, with especially sharp increases observed in economically developed regions such as Zhejiang Province (CRC incidence rose by 41.3% from 2015 to 2022)^[2,3]^. Screening has proven effective in reducing CRC incidence and mortality^[4,5]^, yet China’s screening coverage remains around 3%, far below that of countries like Denmark (75%), South Korea (64%), and the US (72%)^[6,7]^. This underutilization exacerbates the CRC burden, necessitating improved screening strategies.

**HIGHLIGHTS**

- This study presents the first province-wide, population-based evaluation of a risk-stratified colorectal cancer (CRC) screening program in China.
- The province-wide, full-coverage implementation of the RF-FIT strategy (risk score + FIT) is both effective and cost-effective, improving detection of CRC and advanced adenomas while reducing unnecessary colonoscopies.
- Annual screening initiated at age 40–44 yields the greatest quality-adjusted life-year gains.
- Findings support expanded CRC screening coverage in resource-rich provinces in China, offering scalable insights for high-burden settings globally.

Global guidelines recommend stool-based tests like fecal immunochemical testing (FIT) or colonoscopy for CRC screening^[8,9]^. However, given China’s large population and limited health care resources, widespread implementation of colonoscopy-based screening is impractical. Moreover, colonoscopy overuse in average-risk populations can be inefficient and potentially harmful, as it contributes to waste of limited endoscopic resources, avoidable health care costs, and increases the risk of procedure-related complications (e.g., bleeding, perforation, and adverse reactions to sedation)^[10,11]^. Recent studies have shown that risk-adapted screening – combining risk prediction models with FIT – can enhance efficiency by identifying high-risk individuals while reducing colonoscopy load^[12–14]^. While early evidence suggests the potential effectiveness of risk-based screening approaches, comprehensive assessments remain lacking, particularly regarding their scalability and applicability across broader populations in China.

Based on China’s first large-scale, province-wide, full-coverage CRC screening program, this study evaluates the benefits, burdens, and cost-effectiveness of a risk-based CRC screening strategy. The objectives are to (1) assess the yield of the risk-based approach in terms of participation rates, detection yields, and deaths averted; (2) evaluate screening costs, colonoscopy burden, and cost-effectiveness; and (3) explore the optimal ages and frequencies. By integrating real-world implementation data with model simulations, this study addresses critical evidence gaps and provides actionable insights to guide policy decisions in China and other regions facing similar health care challenges.

This article is compliant with the TITAN Guidelines 2025^[15]^.

## Methods

### Study design and participants

**Figure 1.**
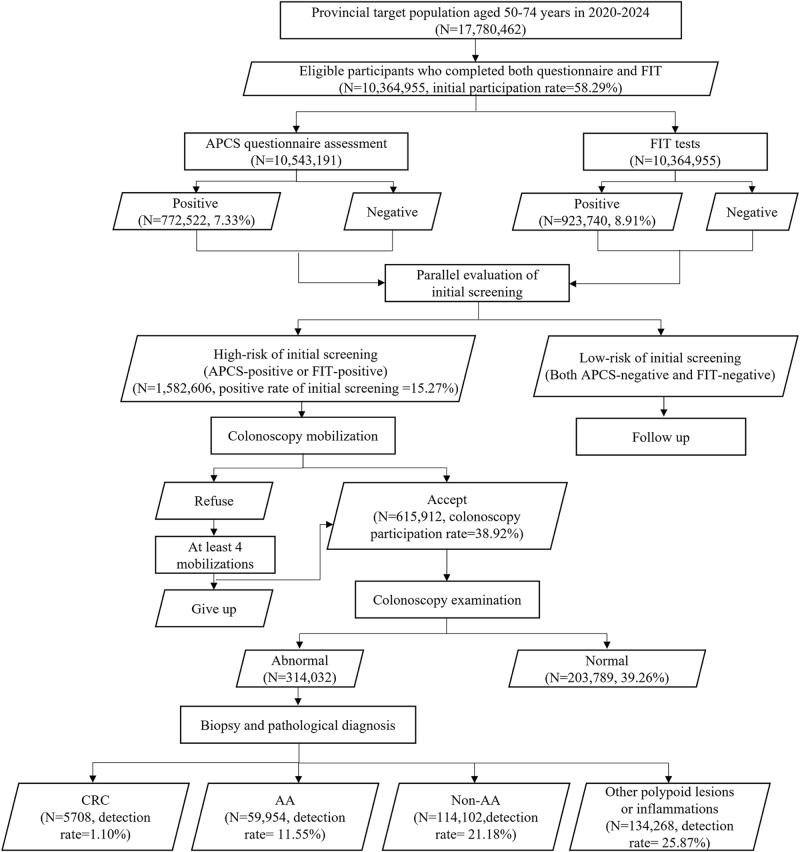
The flowchart of the screening programme.

The Province-wide Colorectal Cancer Screening Program with Full Coverage (PCCSP) was implemented in Zhejiang Province from 2020 to 2024, targeting approximately 18 million residents. Full coverage was defined as the complete mobilization of the eligible target population within Zhejiang Province, rather than participation rate. Inclusion criteria: residents aged 50–74; willing and able to provide written informed consent. Exclusion criteria: diagnosed with colorectal malignancy; presence of severe intellectual disability or communication disorders; suffering from serious cardiovascular, cerebrovascular, pulmonary diseases, significant hepatic, or renal dysfunction; and underwent colonoscopy within the past year. The flowchart of the screening process is presented in Figure 1.

Initial screening serves as a risk stratification strategy for average-risk individuals, integrating the risk factor-based Revised Optimized Asia-Pacific Colorectal Screening Score (APCS)^[16–18]^ questionnaire with FIT, collectively referred as the RF-FIT strategy. Individuals with APCS-positive or FIT-positive results were identified as high risk for initial screening. Those high-risk individuals were referred to undergo colonoscopy following the China guidelines^[19]^. Individuals with negative results on both the APCS and FIT are subject to annual passive follow-up, during which their health status is monitored through linkage with electronic health records, cancer registries, and mortality databases.

We used the modified APCS score, which comprises five CRC-related risk factors, including age, sex, cigarette smoking, body mass index, history of familial adenomatous polyps among first-degree relatives, and history of colonic polyps. Participants with a score ≥5 were defined as APCS positive and those with a score <5 were defined as APCS negative. Individuals with APCS-positive were considered high risk and recommended for colonoscopy. The details of the APCS risk score calculation are described in Supplemental Digital Content Table S1, available at: http://links.lww.com/JS9/G718.

For FIT, we employed the OC-Sensor FIT assay (Eiken Chemical Co., Ltd., Tokyo, Japan), a latex agglutination turbidimetric method for quantifying fecal hemoglobin. The positive detection threshold was set at 20 μg Hb/g feces (equivalent to 100 ng Hb/mL buffer). The colloidal gold method provides qualitative results^[20]^, which require residents to use two specimen collection tubes, and a positive result in either test is considered a positive FIT test. Individuals with FIT-positive were scheduled for a subsequent diagnostic colonoscopy.

Quality assurance was maintained via face-to-face interviews, electronic data entry with compliance checks, and periodic audits. http://links.lww.com/JS9/G586 All patients provided written informed consent before participating in the study. This study is reported in line with the Consolidated Health Economic Evaluation Reporting Standards (CHEERS) 2022 Statement (Supporting information: Checklist)^[21]^. This article is compliant with the TITAN Guidelines 2025^[15]^. No AI was used in the research and manuscript development.

### Mathematical model construction and validation

A Markov model simulated CRC progression in a cohort of 100 000 individuals, evaluating seven initial screening ages (40–44, 45–49, 50–54, 55–59, 60–64, 65–69, and 70–74 years) and six frequencies (once per lifetime, every 10 years, 5 years, 3 years, 2 years, and annually), with routine care (no specific population-level screening) as the reference. For each initial screening age, a closed cohort of 100 000 participants with a mean age of 42, 47, 52, 57, 62, 67, or 72 years was assumed to enter the model. While the PCCSP residents aged 50–74 years, the model additionally simulated hypothetical cohorts starting at age 40 to evaluate the potential benefits and cost-effectiveness of earlier screening initiation in light of the rising incidence of early-onset CRC in China^[22,23]^. Key model assumptions are summarized in Supplemental Digital Content Table S2, available at: http://links.lww.com/JS9/G718.

**Figure 2.**
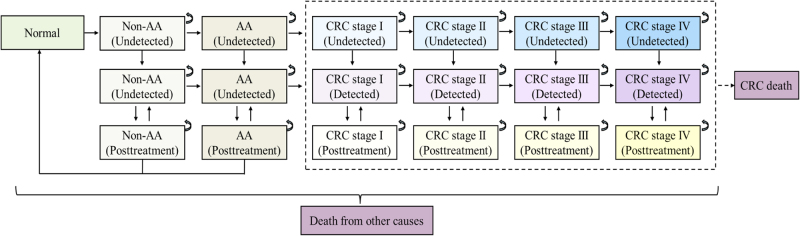
Structure of Markov process model.

The model consists of 21 health states (Fig. 2), following the adenoma-carcinoma sequence, which accounts for over 95% of cases in the Asian population and has been the most extensively studied^[24,25]^. The model accounts for recurrence, false positives, and post-treatment follow-up^[26]^. As individuals age, the normal epithelium may progress to non-advanced adenomas (NAA), and then develop into advanced adenomas (AA) – defined as at least one adenoma >10 mm in size, one with villous components, or high-grade intraepithelial neoplasia. CRC was coded as C18-C21 according to the International Classification of Diseases 10th Revision. Disease progression is irreversible. Advanced neoplasms (ANs) were defined as AA plus CRC only and did not include sessile serrated lesions (SSLs) or other types of polyps. The model was run in annual cycles, terminating when the mean cohort age reached 82 years (the life expectancy in Zhejiang in 2023), with a half-cycle correction applied. We calibrated model parameters based on the age-specific initial distribution of CRC-related health states among the screened population of PCCSP.

In internal validation, five experts in different fields including clinical, epidemiology, and health economics were invited to confirm the face validity of the model. Two team members independently examined the model programming and calculation results and gave a unanimous judgment. Model outputs about the tendency of each pathological grade proportion were checked with the characteristics of the natural history of CRC. For example, the proportions decreased with the severity of the disease in each age group, and the proportions of each CRC pathological grade increased with age. We also simulated each parameter change in a broad range to determine whether the direction and magnitude of model outputs behave as expected. In extensive validation, the observed epidemiological data on incidence and mortality reported by cancer registries in Zhejiang in 2023 were used as reference^[3]^. A closed cohort of people aged 40−44 years was assumed to enter the model without screening intervention, and model projected outputs. The projected age-specific CRC incidence and mortality rates were compared with the references. Root Mean Square Error (RMSE) is a commonly used metric for measuring the discrepancy between predicted and observed values, which is the square root of the mean of the squared differences between predicted and actual values. A smaller RMSE indicates more accurate predictions. The projected and observed CRC incidence showed a good fit (*R*^2^ = 0.964, RMSE = 15.815), and the mortality curves also demonstrated a high level of agreement (*R*^2^ = 0.926, RMSE = 15.530). The validation results were shown in Supplemental Digital Content Figure S1, available at: http://links.lww.com/JS9/G718.

### Model parameters

Estimates of parameters used in the model are presented in Table 1. Participation rate of initial screening (58.29%, range: 50.52%–59.67%) and colonoscopy compliance (38.92%, range: 26.90%–52.90%) were derived from PCCSP data. Supplemental Digital Content Table S3, available at: http://links.lww.com/JS9/G718 presents the age-specific initial probability distribution of CRC-related health states from PCCSP. Screening and medical costs were obtained from a survey in the PCCSP (Supplemental Digital Content Table S4–S5, available at: http://links.lww.com/JS9/G718). The accurate true positive and true negative rates for APCS, FIT, and colonoscopy were drawn from previous studies: initial screening: sensitivity for NAA/AA: 0.5476 (0.3530–0.6750); sensitivity for CRC: 0.7778 (0.4000–0.9510); and specificity: 0.7170 (0.5000–0.9000)^[27,28]^. Colonoscopy: sensitivity for NAA: 0.85 (0.50–1.00); sensitivity for AA: 0.95 (0.80–1.00); sensitivity for CRC: 1.00 (0.95–1.00); and specificity: 0.95 (0.95–1.00)^[29,30]^. We assumed that CRC patients exhibit a significant decline in health-related quality of life scores following diagnosis and post-treatment. Self-initiated examinations, treatment compliance, recurrence, utility, and other estimates of parameters were mainly obtained from published literature^[31–40].^ The 5-year survival rate for CRC was converted into an annual mortality rate in the model, using the formula: annual mortality rate = 1 – (5-year survival rate)^1/5^. Data on age-specific all-cause mortality were sourced from the China Population and Employment Statistical Yearbook 2022^[41]^. Costs and quality-adjusted life-year (QALYs) were discounted at a rate of 3% (range, 0%–5%) and presented as 2023 values^[42]^.

**Table 1:** Key parameters.

Parameter	Base-case	95% UI	Distribution	Source
Costs, *$*				
Screening costs				
Preparation cost^a^	13 572	6786–20 358	Triangular	PCCSP
Per-person cost of initial screening^b^	3.26	1.63–4.89	Triangular	PCCSP
Per-person cost of fine screening^c^	95.80	47.90–143.70	Triangular	PCCSP
Treatment costs				
NAA	706	353–1059	Gamma	PCCSP
AA	1185	593–1778	Gamma	PCCSP
CRC stage I	6038	3019–9057	Gamma	PCCSP
CRC stage III	8821	4410–13 231	Gamma	PCCSP
CRC stage III	9468	4734–14 202	Gamma	PCCSP
CRC stage IV	10 265	5132–15 397	Gamma	PCCSP
Diagnose costs of colorectal lesions^d^	86.43	43.22–129.65	Gamma	PCCSP
Follow-up of colorectal lesions^e^	86.43	43.22–129.65	Gamma	PCCSP
Initial screening^f^				
Participation rate	0.5829	0.5052–0.5967	Triangular	PCCSP
Sensitivity for NAA/AA	0.5476	0.3530–0.6750	Triangular	^[27]^
Sensitivity for CRC Specificity	0.7778	0.4000–0.9510	Triangular	^[27]^
	0.7170	0.5000–0.9000	Triangular	^[28]^
Colonoscopy				
Compliance with colonoscopy	0.3892	0.2690–0.5290	Triangular	PCCSP
Sensitivity for NAA	0.85	0.50–1.00	Triangular	^[29]^
Sensitivity for AA	0.95	0.80–1.00	Triangular	^[29]^
Sensitivity for CRC	1.00	0.95–1.00	Triangular	^[29]^
Specificity	0.95	0.95–1.00	Triangular	^[30]^
Annual transition probability				
From normal to NAA				
40–49 years	0.0147	0.0073–0.0220	Uniform	Calibration
50–54 years	0.0182	0.0091–0.0273	Uniform	Calibration
55–59 years	0.0192	0.0096–0.0288	Uniform	Calibration
60–64 years	0.0244	0.0122–0.0366	Uniform	Calibration
65–69 years	0.0261	0.0131–0.0392	Uniform	
				Calibration
70–74 years	0.0412	0.0206–0.0618	Uniform	Calibration
75–79 years	0.0525	0.0263–0.0788	Uniform	Calibration
80+ years	0.0606	0.0303–0.0909	Uniform	Calibration
From NAA to AA				
40–49 years	0.0837	0.0418–0.1255	Uniform	Calibration
50–54 years	0.1093	0.0547–0.1640	Uniform	Calibration
55–59 years	0.1262	0.0631–0.1892	Uniform	Calibration
60–64 years	0.1395	0.0698–0.2093	Uniform	Calibration
65–69 years	0.1735	0.0868–0.2603	Uniform	Calibration
70–74 years	0.2391	0.1196–0.3587	Uniform	Calibration
75–79 years	0.2402	0.1201–0.3603	Uniform	Calibration
80+ years	0.2445	0.1222–0.3668	Uniform	Calibration
From AA to CRC stage I				
40–49 years	0.0158	0.0079–0.0237	Uniform	Calibration
50–54 years	0.0164	0.0082–0.0246	Uniform	Calibration
55–59 years	0.0171	0.0086–0.0257	Uniform	Calibration
60–64 years	0.0232	0.0116–0.0348	Uniform	Calibration
65–69 years	0.0260	0.0130–0.0390	Uniform	Calibration
70–74 years	0.0298	0.0149–0.0447	Uniform	Calibration
75–79 years	0.0302	0.0151–0.0454	Uniform	Calibration
80+ years	0.0370	0.0185–0.0555	Uniform	Calibration
From stage I to stage II	0.30	0.10–0.50	Beta	^[31]^
From stage II to stage III	0.45	0.20–0.90	Beta	^[31]^
From stage III to stage IV	0.50	0.30–1.00	Beta	^[31]^
Symptom-detected rate				
NAA	0.145	0.00–0.20	Beta	^[32]^
AA	0.145	0.00–0.20	Beta	^[32]^
CRC stage I	0.07	0.00–0.30	Beta	^[33]^
CRC stage II	0.25	0.20–0.50	Beta	^[33]^
CRC stage III	0.55	0.50–0.70	Beta	^[33]^
CRC stage IV	0.85	0.70–1.00	Beta	^[33]^
Treatment compliance				
NAA	0.60	0.50–0.70	Beta	^[34]^
AA	0.80	0.70–0.90	Beta	^[34]^
CRC stage I	0.95	0.85–1.00	Beta	^[34]^
CRC stage II	0.95	0.85–1.00	Beta	^[34]^
CRC stage III	0.95	0.85–1.00	Beta	^[34]^
CRC stage IV	0.95	0.85–1.00	Beta	^[34]^
Annual recurrence probability after treatment				
NAA	0.0663	0.0300–0.0900	Uniform	^[35]^
AA	0.1071	0.0500–0.1500	Uniform	^[35]^
CRC stage I	0.0252	0.0200–0.0300	Uniform	^[36]^
CRC stage II	0.0389	0.0300–0.0500	Uniform	^[36]^
CRC stage III	0.0715	0.0600–0.0800	Uniform	^[36]^
CRC stage IV	0.1176	0.0800–0.1500	Uniform	Assumption
Utility scores				
General population/NAA	40–54: 0.957	0.900–1.000	Triangular	^[34,37]^
	55–59: 0.955			
	60–64: 0.957			
	65–82: 0.943			
AA	0.83	0.80–0.90	Beta	^[38]^
CRC stage I	0.74	0.70–0.80	Beta	^[38]^
CRC stage II	0.74	0.60–0.80	Beta	^[38]^
CRC stage III	0.67	0.50–0.80	Beta	^[38]^
CRC stage IV	0.25	0.10–0.60	Beta	^[38]^
5-year survival rate				
CRC stage I	0.916	0.881–0.975	Triangular	^[39]^
CRC stage II	0.848	0.774–0.951	Triangular	^[39]^
CRC stage III	0.689	0.590–0.774	Triangular	^[39]^
CRC stage IV	0.188	0.169–0.237	Triangular	^[39]^
Background mortality				
40–44 years	0.00109			^[41]^
45–49 years	0.00226			
50–54 years	0.00349			
55–59 years	0.00606			
60–64 years	0.00875			
65–69 years	0.01530			
70–74 years	0.02443			
75–79 years	0.04291			
80–82 years	0.06802			
Discount rate	0.03	0.00–0.05		^[42]^

AA, advanced adenomas; CRC, colorectal cancer; Fully-covered Colorectal Cancer Screening Programme; NAA, non-advanced adenomas; PCCSP, The Provincial-wide; UI, uncertainty interval.

^a^ The preparation costs covered expenses for staff training, public health education, and the construction of the screening system. All costs were reported in US dollars (USD).

^b^ The initial screening costs covered expenses for mobilization and organization, questionnaire surveys, FIT tests, and mobilization for colonoscopy.

^c^ Fine screening costs included pre-colonoscopy preparation (e.g., electrocardiogram and blood tests), colonoscopy, and pathological examination.

^d^ Including pre-colonoscopy preparation, colonoscopy, and pathological examination.

^e^ Follow-up costs include pre-colonoscopy preparation, colonoscopy, and pathological examination.

^f^ Initial screening included APCS questionnaire combined with FIT. Individuals with APCS ≥5 or FIT-positive were identified as high-risk of initial screening. The sensitivity and specificity of initial screening refers to the performance metrics of APCS questionnaire combined with FIT strategy.

### Costing analysis

Screening costs were surveyed across 11 cities and 93 counties (districts) of Zhejiang Province. The costs encompassed the preparation costs, initial screening costs, and fine screening costs. The initial screening costs covered expenses for mobilization and organization, questionnaire surveys, FIT tests, and mobilization for colonoscopy. Fine screening costs included pre-colonoscopy preparation (e.g., electrocardiogram and blood tests), colonoscopy, and pathology fees. All costs are surveyed using Chinese Yuan (CNY) and are converted and presented as U.S. dollars (1 US*$* = 7.0467 CNY) in the study. The total screening cost was *$*95 231 764 from 2020 to 2024 (Supplemental Digital Content Table S4, available at: http://links.lww.com/JS9/G718). The per-person cost of initial screening and fine screening was *$*3.26 and *$*95.80, respectively.

From the perspective of health care system, the per-person medical cost was obtained using Delphi interviews and the medical records extraction approach in hospitals, which refers to the direct medical expenses incurred throughout the entire course of CRC-related conditions. These include outpatient costs, medication, therapeutic costs, imaging, and laboratory tests, and also account for the costs associated with disease recurrence. We consulted 548 clinicians from 231 hospitals to obtain the medical costs of NAA and AA. The average medical costs per NAA and AA were *$*706 and *$*1185, respectively. The medical cost of CRC was extracted from the medical records of 16 hospitals, involving 2377 CRC patients. The average medical costs per CRC stage I, II, III, and IV were *$*6038, *$*8821, *$*9468, and *$*10 265, respectively (Supplemental Digital Content Table S5, available at: http://links.lww.com/JS9/G718).

### Yield, required resources, and cost-effectiveness of screening

The outcomes included yield, burden, and cost-effectiveness. (1) The screening yield included initial participation rate, colonoscopy participation rate, detection rate, and death averted. (2) Several resource-consumption metrics were calculated to evaluate affordability, including the number of colonoscopies to detect one lesion, costs to detect one lesion, and the number needed to screen initially and clinically per CRC death averted. (3) Cost-effectiveness was assessed by incremental cost-effectiveness ratios (ICERs), benchmarked against three times per capita GDP (*$*53 235 in 2023) per QALY gained. We also applied the cost-effectiveness threshold for chronic diseases in China (1.76 times the per capita gross domestic product)^[43]^.

### Sensitivity analyses

We conducted one-way deterministic sensitivity analyses by varying individual parameters across their plausible ranges while holding others constant to assess the impact of key variables. To capture joint parameter uncertainty, we further performed probabilistic sensitivity analysis (PSA) using 10 000 Monte Carlo simulations, in which all model parameters were simultaneously sampled from predefined probability distributions. The results of the PSA were used to calculate the 95% uncertainty intervals of the model results. Each simulation iteration generated an ICER, and the results were summarized using cost-effectiveness acceptability curves (CEACs) to evaluate decision uncertainty under different willingness-to-pay thresholds.

## Results

### Screening yield and required resources

The PCCSP targeted 17 780 462 individuals, with 10 364 955 (58.29%) completing both the questionnaire and FIT. Of these, 1 582 606 (15.27%) screened positive and were invited for colonoscopy, resulting in 615 912 colonoscopies (38.92% compliance). The program detected 114 102 NAAs (detection rate: 21.18%), 59 954 AAs (11.55%), and 5708 CRCs (1.10%; Fig. 1).

Screening yield per 10 000 participants was 43 NAAs, 24 AAs, and 3 CRCs, while yield per 10 000 colonoscopies was 825 NAAs, 450 AAs, and 43 CRCs. The program required 6, 11, and 108 colonoscopies to detect one NAA, AA, and CRC, respectively, at costs of *$*835, *$*1588, and *$*16 684 per lesion. The PCCSP averted 19 026 CRC deaths, with 532 screenings needed to prevent one death (Table 2). Compared to routine care, all screening strategies prevent CRC deaths, with annual screening for the 40–44 age group yielding the highest number of deaths averted (1126 per 10 000 cohort members) and requiring 158 colonoscopies per death prevented (Table 3).

**Table 2:** Efficacy and required medical resources of risk-based colorectal cancer screening in China.

Efficacy and required medical resources	NAA	AA	CRC	AN
Detection (*n*)	114 102	59 954	5708	65 662
Colonoscopies to detect one lesion (*n*)	6	11	108	10
Yield per 10 000 participants (*n*)	43	24	3	26
Yield per 10 000 colonoscopies (*n*)	825	450	43	493
Invitees to detect one lesion (*n*)	233	417	3333	385
Cost to detect one lesion (*$*)	835	1588	16 684	1450
CRC deaths averted	-	-	19 026	-
Number needed to screen initially per CRC death averted	-	-	532	-
Number needed to screen clinically per CRC death averted	-	-	64	-

AA, advanced adenomas; AN, advanced neoplasms, included AA and CRC; CRC, colorectal cancer; NAA, non-advanced adenomas.

Yield per 10 000 participants = 10 000 × participation rate of initial screening × positive rate of initial screening × colonoscopy participation rate × detection rate.

Yield per 10 000 colonoscopies = 10 000 × colonoscopy participation rate × detection rate.

Number needed to screen initially per CRC death averted = number of individuals who completed the initial screening/number of CRC death averted.

Number needed to screen clinically per CRC death averted = number of colonoscopy screening/number of CRC death averted.

**Table 3:** **Deaths averted compared among different strategies by initial screening age among 100 000 cohort^a^**.

Initial screening age, strategy	CRC deaths averted		Number needed to screen clinically per CRC death averted	
	Vs no screening	Vs the next most effective strategy^b^	Vs no screening	Vs the next most effective strategy^b^
40–44 y				
No screening	Ref	Ref	Ref	Ref
Screening once per lifetime	65	65	103	103
Screening every 10 years	280	215	77	69
Screening every 5 years	459	179	82	91
Screening every 3 years	647	188	92	115
Screening every 2 years	843	196	107	157
Screening every years	1126	283	158	311
45–49 years				
No screening	Ref	Ref	Ref	Ref
Screening once per lifetime	71	71	95	95
Screening every 10 years	234	163	74	65
Screening every 5 years	424	190	77	82
Screening every 3 years	612	188	87	110
Screening every 2 years	760	148	99	149
Screening every years	1027	267	147	283
50–59 years				
No screening	Ref	Ref	Ref	Ref
Screening once per lifetime	94	94	73	73
Screening every 10 years	251	157	67	63
Screening every 5 years	395	144	71	77
Screening every 3 years	559	164	79	97
Screening every 2 years	713	154	91	135
Screening every year	946	233	131	255
55–59 years				
No screening	Ref	Ref	Ref	Ref
Screening once per lifetime	108	108	64	64
Screening every 10 years	200	92	62	59
Screening every 5 years	347	147	67	73
Screening every 3 years	475	128	72	88
Screening every 2 years	608	133	83	121
Screening every year	828	220	119	217
60-64 years				
No screening	Ref	Ref	Ref	Ref
Screening once per lifetime	120	120	58	58
Screening every 10 years	194	74	63	70
Screening every 5 years	279	85	65	70
Screening every 3 years	396	117	73	93
Screening every 2 years	493	97	82	115
Screening every year	665	172	110	190
65–69 y				
No screening	Ref	Ref	Ref	Ref
Screening once per lifetime	121	121	58	58
Screening every 5 years	200	79	65	75
Screening every 3 years	270	70	70	85
Screening every 2 years	328	58	76	104
Screening every year	478	150	99	149
70–74 y				
No screening	Ref	Ref	Ref	Ref
Screening once per lifetime	104	104	68	68
Screening every 2 years	163	59	83	108
Screening every year	219	56	91	113

^a^ QALYs, costs, and ICERs are expressed as the values in 2023. All costs were reported in US dollars (USD).

^b^ Compared with the next most effective strategy at the same initial screening age.

Sex-stratified analyses revealed clear heterogeneity in CRC screening outcomes (Supplemental Digital Content Table S6, available at: http://links.lww.com/JS9/G718). Women showed higher participation (62.9% vs. 53.3%) and colonoscopy compliance (43.3% vs. 35.9%), whereas men had substantially higher detection rates for NAA, AA, and CRC. In simulated cohorts, men consistently yielded more detected lesions and greater numbers of CRC deaths prevented compared with women. Efficiency analyses further indicated that men required fewer invitations and colonoscopies per lesion detected (Supplemental Digital Content Table S7, available at: http://links.lww.com/JS9/G718).

### Base-case cost-effectiveness

**Figure 3.**
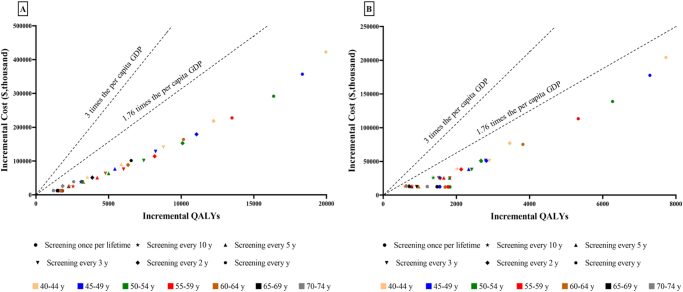
Incremental cost-effectiveness ratio of all screening strategies by initial screening age. (A) Compared with no screening. (B) Competing with each other.

Compared to routine care, all screening strategies were cost-effective, regardless of the starting age or screening frequency, adding 1206–19 955 QALYs and increasing costs by *$*12 127 to *$*422 588, yielding ICERs of *$*6718 to *$*21 177 per QALY, all below three times the per capita GDP (Table 4, Fig. 3). Comparing each screening strategy to the next most effective one yielded an additional 601–7726 QALYs, with incremental costs ranging from $12 127 to $204 077 for a cohort of 100 000 participants over a lifetime. The corresponding ICERs were $185 to $11 106 per QALY, all lower than three times the per capita GDP. Annual RF-FIT screening, particularly for ages 40–44, was the most cost-effective, yielding 19 955 incremental QALYs (Table 4, Fig. 3). Sex-stratified analysis indicated that male-targeted screening strategies generated greater health gains at lower costs (Supplemental Digital Content Table S8, available at: http://links.lww.com/JS9/G718).

**Table 4:** **Cost-effectiveness compared among different strategies by initial screening age among 100 000 cohort^a^**.

Initial screening age, strategy	QALYs	Incremental QALYs		Cost ($, thousand)	Incremental cost (*$*, thousand)		ICER (*$*/QALY)	
		Vs no screening	Vs the next most effective strategy^b^		Vs no screening	Vs the next most effective strategy^b^	Vs no screening	Vs the next most effective strategy^b^
40–44 years								
No screening	2 064 861	Ref	Ref	105 827	Ref	Ref	Ref	Ref
Screening once per lifetime	2 066 380	1519	1519	118 197	12 370	12 370	8144	8144
Screening every 10 years	2 068 402	3541	2022	157 206	51 379	39 009	14 509	19 289
Screening every 5 years	2 070 731	5870	2329	195 957	90 129	38 751	15 354	16 640
Screening every 3 years	2 073 634	8773	2903	247 221	141 394	51 264	16 118	17 661
Screening every 2 years	2 077 090	12 229	3457	324 338	218 510	77 117	17 868	22 310
Screening every years	2 084 817	19 955	7726	528 415	422 588	204 077	21 177	26 414
45–49 years								
No screening	1 898 860	Ref	Ref	102 463	Ref	Ref	Ref	Ref
Screening once per lifetime	1 900 407	1546	1546	114 991	12 529	12 529	8102	8102
Screening every 10 years	1 901 953	3093	1547	140 916	38 453	25 925	12 433	16 762
Screening every 5 years	1 904 293	5432	2339	179 431	76 968	38 515	14 169	16 465
Screening every 3 years	1 907 096	8236	2804	230 810	128 348	51 380	15 584	18 327
Screening every 2 years	1 909 915	11 055	2819	281 726	179 264	50 916	16 216	18 062
Screening every years	1 917 203	18 342	7287	459 372	356 910	177 646	19 459	24 377
50–59 years								
No screening	1 708 991	Ref	Ref	98 999	Ref	Ref	Ref	Ref
Screening once per lifetime	1 710 812	1821	1821	111 232	12 233	12 233	6718	6718
Screening every 10 years	1 712 179	3188	1367	137 158	38 160	25 927	11 970	18 966
Screening every 5 years	1 713 993	5002	1815	162 712	63 713	25 553	12 736	14 083
Screening every 3 y	1 716 414	7423	2420	200 786	101 787	38 074	13 713	15 730
Screening every 2 years	1 719 086	10 096	2673	251 841	152 842	51 055	15 140	19 103
Screening every year	1 725 353	16 362	6267	390 775	291 776	138 934	17 832	22 171
55–59 years								
No screening	1 499 103	Ref	Ref	91 176	Ref	Ref	Ref	Ref
Screening once per lifetime	1 500 874	1771	1771	103 523	12 347	12 347	6971	6971
Screening every 10 years	1 501 662	2559	788	116 451	25 276	12 929	9877	16 409
Screening every 5 years	1 503 318	4216	1657	141 995	50 819	25 544	12 055	15 418
Screening every 3 years	1 505 139	6036	1820	167 151	75 976	25 156	12 587	13 820
Screening every 2 years	1 507 270	8168	2132	205 461	114 285	38 310	13 993	17 972
Screening every year	1 512 601	13 498	5331	318 771	227 595	113 310	16 861	21 255
60–64 years								
No screening	1 266 933	Ref	Ref	79 556	Ref	Ref	Ref	Ref
Screening once per lifetime	1 268 627	1695	1695	91 684	12 127	12 127	7156	7156
Screening every 10 years	1 269 229	2296	601	104 764	25 208	13 080	10 980	21 758
Screening every 5 years	1 270 199	3267	971	117 450	37 894	12 686	11 600	13 067
Screening every 3 years	1 271 715	4782	1515	143 100	63 544	25 650	13 289	16 928
Screening every 2 years	1 273 272	6339	1557	168 229	88 673	25 129	13 989	16 140
Screening every year	1 277 088	10 156	3817	243 514	163 957	75 285	16 145	19 725
65–69 years								
No screening	1 010 441	Ref	Ref	67 551	Ref	Ref	Ref	Ref
Screening once per lifetime	1 011 915	1474	1474	80 082	12 531	12 531	8501	8501
Screening every 5 years	1 012 697	2256	782	92 989	25 438	12 907	11 277	16 512
Screening every 3 years	1 013 614	3173	917	105 454	37 904	12 465	11 947	13 593
Screening every 2 years	1 014 320	3879	706	118 598	51 047	13 143	13 160	18 615
Screening every year	1 016 997	6555	2677	169 045	101 495	50 448	15 483	18 848
70–74 years								
No screening	727 150	Ref	Ref	50 040	Ref	Ref	Ref	Ref
Screening once per lifetime	728 356	1206	1206	62 789	12 749	12 749	10 574	10 574
Screening every 2 years	728 990	1839	634	76 242	26 202	13 453	14 244	21 229
Screening every year	729 747	2597	758	89 150	39 110	12 908	15 059	17 035

ICER, incremental cost-effectiveness ratio; QALY, quality-adjusted life-year.

^a^ QALYs, costs, and ICERs are expressed as the values in 2023. All costs were reported in US dollars (USD).

^b^ Compared with the next most effective strategy at the same initial screening age.

### One-way and PSAs

**Figure 4.**
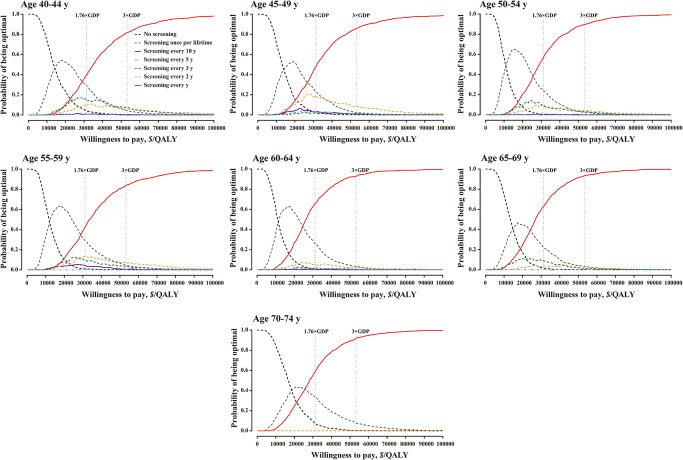
Cost-effectiveness acceptability curves of all strategies competing with each other.

**Figure 5.**
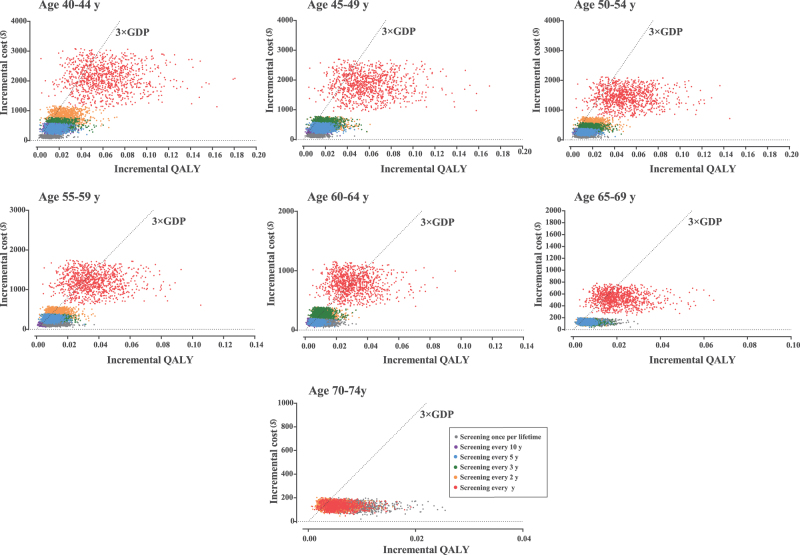
Probabilistic sensitivity analysis of all strategies competing with each other.

One-way sensitivity analyses on participation rate of initial screening, colonoscopy compliance, transition probabilities, screening costs, treatment costs, and discount rates consistently confirmed the cost-effectiveness of all strategies, with ICER remaining below three times the per capita GDP. Annual screening for ages 40–44 consistently emerged as the optimal strategy (Supplemental Digital Content Table S9, available at: http://links.lww.com/JS9/G718). PSA further validated these findings, showing a >90% probability of cost-effectiveness at a willingness-to-pay threshold of three times the per capita GDP (Supplemental Digital Content Figures S2–S3, available at: http://links.lww.com/JS9/G718). Annual screening maintained dominance across all age groups (Fig. 4), with an 81.9%–93.2% probability of being optimal (Fig. 5).

We conducted additional one-way sensitivity analyses in which the participation rate for initial screening and the compliance rate for colonoscopy were each varied across a broader range of plausible values (30%, 40%, 50%, 60%, 70%, and 80%; Supplemental Digital Content Tables S10–S11, available at: http://links.lww.com/JS9/G718); the results indicate that although varying participation or compliance rates influences the absolute magnitude of cost-effectiveness outcomes, these changes do not alter the overall conclusions regarding the optimal screening strategies. Additionally, we incorporated frequency-dependent adherence decay into sensitivity analyses, under the assumption that both participation and colonoscopy compliance gradually decline as screening intensity increases. The results confirmed that ICERs for high-frequency strategies increased as adherence declined; however, all strategies remained cost-effective within the willingness-to-pay threshold (Supplemental Digital Content Table S12, available at: http://links.lww.com/JS9/G718).

To assess potential impact of colonoscopy-related complications, we conducted a sensitivity analysis. Results showed only marginal changes in cost-effectiveness estimates, with all ICERs remaining below three times per capita GDP (Supplemental Digital Content Table S13, available at: http://links.lww.com/JS9/G718).

## Discussion

This study represents a landmark in CRC screening in China, being the first province-wide, full-coverage initiative to evaluate the benefits, burdens, and cost-effectiveness of a risk-based strategy. By integrating real-world data with rigorous modeling, we demonstrate that a risk-based screening approach is not only feasible and affordable but also highly cost-effective compared to routine care. These findings provide critical evidence for scaling up CRC screening across China, offering valuable insights for national policy decisions and future investment in preventive health initiatives.

Globally, organized screening programs based on FIT or colonoscopy have been widely adopted^[9,44,45]^. However, studies indicate that over 24% of colonoscopies in average-risk populations may be overused^[10,46]^, leading to resource inefficiencies. A risk-adapted approach, prioritizing high-risk individuals, is particularly beneficial in regions with limited health care resources^[14,47]^. This aligns with the Asia Pacific Working Group recommendation and Chinese guidelines (strong recommendation)^[19,48]^, which recommend combining risk assessment questionnaires with FIT as an initial screening step, followed by colonoscopy for high-risk individuals. This combined approach enhances detection efficiency by leveraging risk scores to identify high-risk populations and FIT to improve lesion detection^[49,50]^.

Our cost-effectiveness analysis reveals that the RF-FIT strategy is both effective and economically viable. It achieved a 12.21% detection rate for AN, significantly outperforming FIT-only (2.17%) and questionnaire-only approach (2.35%)^[18]^. This result is consistent with previous studies demonstrating that RF-FIT increases colonoscopy uptake (~7%) and improves detection rates^[47]^. Additionally, RF-FIT reduces colonoscopy burden and costs, requiring 11 and 108 colonoscopies per detected AA and CRC, with associated costs of $1588 and $16 684 per detected AA and CRC. These results represent a substantial improvement over previous questionnaire-based screening programs in urban China, which required 33 and 394 colonoscopies per detected AA and CRC, respectively^[51]^. Furthermore, compared to national and regional RF-FIT programs^[47,49]^, our study demonstrated higher lesion detection rates, fewer colonoscopies needed, and lower associated costs, reinforcing the feasibility and cost-effectiveness of province-wide RF-FIT screening. However, further investigation is needed to assess the long-term effectiveness and optimal screening intervals.

In addition to comparisons with no screening, we evaluated the performance of the RF-FIT strategy against conventional single-modality approaches (Supplemental Digital Content Table S14, available at: http://links.lww.com/JS9/G718). Compared with FIT-only, the RF-FIT strategy combines a non-invasive risk assessment questionnaire with FIT. This approach captures high-risk, FIT-negative individuals who would otherwise be missed. This complementary approach increases lesion detection and overall health gains, although it modestly raises follow-up colonoscopy numbers and costs^[52]^. Despite higher incremental costs, the ICER remains within commonly accepted willingness-to-pay thresholds, indicating cost-effectiveness. Compared with colonoscopy-only, RF-FIT has slightly lower diagnostic sensitivity but substantially higher participation and adherence due to its non-invasive nature^[53]^. Importantly, RF-FIT dramatically reduces the number of colonoscopies required, concentrating resources on high-risk individuals while maintaining population-level health benefits. These findings suggest that RF-FIT achieves a favorable balance between effectiveness, feasibility, and economic efficiency, making it particularly suitable for large-scale CRC screening programs in resource-constrained settings, where universal colonoscopy is impractical and FIT-only strategies may miss high-risk cases.

Our sex-stratified analyses underscore substantial heterogeneity in CRC screening outcomes. Despite higher participation and compliance among women, men exhibited markedly greater lesion detection rates and absolute health gains, with screening preventing more CRC deaths in men than in women. These findings highlight men as a paradoxical “high-risk but low-participation” subgroup, underscoring the importance of incorporating sex into risk-adapted screening strategies. From a policy perspective, our sex-specific findings suggest that in settings with constrained colonoscopy capacity or limited health care resources, prioritizing earlier or more intensified screening among men may yield greater population-level health benefits and lower opportunity costs. To enhance the feasibility and impact of such a sex-differentiated approach, tailored interventions are warranted to improve screening participation and adherence among men. These could include the development of targeted health communication strategies that address men’s perceptions of risk and prevention – for example, by emphasizing that timely screening effectively reduces the risk of advanced disease and mortality. Additionally, offering more flexible screening schedules, such as evening or weekend appointments, could better accommodate men’s work and lifestyle patterns. Further consideration could also be given to integrating CRC screening into health care settings that men frequently access, such as urology clinics, chronic disease management visits, or workplace health check-up programs. Adopting this risk-stratified and gender-informed approach to screening allocation could optimize both the health outcomes and cost-effectiveness of organized CRC screening initiatives. Nevertheless, multi-center studies are needed to confirm whether similar sex heterogeneity is generalizable across diverse populations.

Our findings support annual RF-FIT screening for individuals aged 40–74, with the greatest benefits observed for those starting at ages 40–44. This aligns with national guidelines and reflects the earlier onset of CRC in China compared to Western populations^[19,48]^. While the current PCCSP offers free screening for individuals aged 50–74 at 5-year intervals, expanding to ages 40–74 with annual screening could enhance effectiveness. However, adjustments must consider real-world constraints, including financial sustainability, colonoscopy capacity, and health care staff workload. Refining screening strategies to maximize health outcomes while optimizing resource allocation is critical. The discrepancy between the observed screening cohort (aged 50–74 years) and the younger modeled cohorts reflects the design of our extended scenario analysis. This approach allows exploration of the potential benefits of earlier screening without altering the empirical PCCSP data, thereby improving the generalizability of the findings for policy considerations.

Screening coverage remains a key determinant of program success. A systematic review that included CRC screening coverage data from 33 countries (22 in Europe, 4 in the Americas, 6 in Asia, and 1 in Oceania)^[54]^ indicates that Finland had the highest screening coverage rate at 79.4% in 2021, while China had the lowest at 1.0% in 2020, far below the median screening coverage rate of 38.0% across these countries. A recent modeling study suggested that increasing screening coverage (at full coverage) could potentially reduce CRC incidence by 38.2% and mortality by 43.2%^[25]^. However, implementation challenges persist due to resource limitations and health disparities. Given the significant differences in CRC incidence and economic development levels across China’s provinces, a one-size-fits-all screening strategy may not be optimal. Based on our findings, we propose targeted recommendations: For high-incidence provinces with better health care resources, such as those along the eastern coastal regions (similar to Zhejiang), the risk stratification strategy evaluated in this study should be prioritized. For lower-incidence provinces with limited resources in the central and western regions, the initial focus should be on raising public awareness and piloting questionnaire-based screening in urban centers targeting high-risk factors, gradually expanding to rural areas. Additionally, provinces with established screening programs should continuously optimize their localized risk stratification models using available data.

While our model confirms the cost-effectiveness of risk-based annual RF-FIT screening, its real-world success depends heavily on sustained participation. In our cohort, colonoscopy compliance remained below 40%, substantially lower than international benchmarks (the American Multi-Society Task Force’s 80% goal^[55]^). If left unaddressed, this low adherence may limit the scalability and sustainability of population-wide programs. From an implementation perspective, enhancing compliance requires a multifaceted approach. Strategies such as active call-recall systems, primary care provider engagement, mobile screening units, and health education campaigns tailored to local beliefs and barriers could significantly improve participation^[56]^. Moreover, incentive-based interventions and use of digital tools (e.g., SMS reminders, app-based bookings) have shown promise in improving adherence in other health programs and warrant pilot testing in CRC screening.

The success of large-scale cancer screening programs such as the PCCSP hinges on not only clinical and economic effectiveness but also the broader health care financing and delivery context. As a government-led initiative, the PCCSP is primarily funded by provincial resources, with additional subsidies from county governments and basic health insurance schemes to minimize out-of-pocket costs for participants. This multi-source financing model has proven feasible in economically developed regions like Zhejiang and may be replicable in other areas such as Beijing, Shanghai, and Jiangsu. International experiences, particularly from the United States and Japan, further underscore the importance of integrating health insurance to enhance long-term program sustainability. Looking ahead, future strategies should explore diversified funding mechanisms – including commercial insurance and public-private partnerships – to improve the accessibility and affordability of screening services.

However, the economic feasibility and operational scalability of such programs remain highly dependent on regional health care capacity and system-level factors. In less-developed areas of China or in low- and middle-income countries, barriers such as limited colonoscopy availability, uneven provider distribution, and high out-of-pocket expenses may impede effective implementation. Moreover, the cost structure of risk-adapted RF-FIT screening involves multiple components – including risk assessment, FIT kit distribution, diagnostic colonoscopy, pathology services, and follow-up care – which may vary substantially across regions. In parallel, patient-level factors such as screening awareness, cultural attitudes toward invasive procedures, and access to medical facilities also influence participation and uptake. These contextual determinants must be fully considered when designing and scaling up national screening strategies. To ensure equitable and sustainable implementation, future research should prioritize region-specific adaptations, conduct comprehensive budget impact analyses, and evaluate the effectiveness of hybrid reimbursement models that blend public funding, social health insurance, and commercial insurance. Integrating such health systems thinking into the design of screening programs will be essential for achieving national coverage and long-term population health benefits.

Equity considerations are essential for designing sustainable and inclusive CRC screening strategies. In Zhejiang Province, the context of our study, urban–rural and socioeconomic disparities are less pronounced than the national average, due to long-standing health system reforms. County-level medical consortia, standardized health examination programs, and province-wide early detection initiatives have narrowed gaps in access, reflected in the minimal difference in travel time to primary care between urban and rural residents. Comparative analyses between Hangzhou (developed) and Lishui (less developed) further showed broadly comparable outcomes, with higher participation and compliance in Lishui despite lower income levels, suggesting that socioeconomic gradients exert limited influence in this setting. Nevertheless, we acknowledge that nationally, disparities in colonoscopy accessibility, income-related participation, and stage-at-diagnosis by ethnicity remain significant. Future multi-province and individual-level studies are warranted to capture these dimensions and inform equitable resource allocation.

Lifestyle-related risk factors warrant particular attention in shaping the future burden of CRC. Although our baseline analysis partially accounted for these factors through the APCS risk score, the Markov model applied static transition probabilities derived from historical data, limiting its ability to reflect China’s rapidly changing epidemiological landscape (e.g., rising obesity prevalence, dietary transitions, and shifting smoking and drinking patterns). Consequently, our findings should be viewed as conservative estimates of screening effectiveness under current conditions. To address this limitation, we incorporated a scenario analysis that integrated projected changes in smoking, obesity, and alcohol consumption into the model using risk multipliers and population-level prevalence forecasts. The results suggested that as lifestyle risks worsen, CRC incidence and mortality would rise markedly, and even intensive screening could not offset declines in overall population QALYs. These findings underscore the complementary roles of primary prevention (e.g., tobacco control, weight management, and alcohol reduction) and secondary prevention (screening) in mitigating CRC burden. They also highlight that static models may underestimate both future disease burden and the potential benefits of screening. Future research should therefore employ more dynamic approaches, such as microsimulation models incorporating evolving risk factors, to generate more realistic long-term projections.

Emerging screening modalities, such as stool-based and blood-based biomarkers, hold promise for improving early detection. Advanced tools like multitarget stool DNA^[57]^ and RNA tests^[58]^ have demonstrated higher sensitivity for AA. Recent studies support the efficacy of blood tests, such as circulating free DNA^[59]^. However, their cost-effectiveness relative to FIT remains uncertain^[60]^. Further validation in large-scale population-based studies is required. While these innovations may complement current approaches, traditional screening remains the foundation of CRC prevention in China.

This study has several key strengths. First, it represents China’s largest province-wide RF-FIT-based CRC screening initiative, covering 93 counties and 236 hospitals, providing robust evidence to guide screening policies. Second, the comprehensive data sources, including real-world observational data from a well-structured program, field surveys, expert consultations, and medical records, ensure a realistic screening evaluation. Third, the model considered recurrence, false positives, and post-treatment follow-up for CRC-related health states, providing a comprehensive economic evaluation. Finally, univariate and PSAs, along with extensive model calibration and validation, confirmed the robustness of the findings.

An important limitation of this study is the assumption of constant adherence across screening strategies. In reality, more intensive approaches – such as annual screening – may face reduced participation and compliance due to greater burden or “screening fatigue.” This could diminish both effectiveness and resource efficiency. To address this, we conducted extensive sensitivity analyses incorporating frequency-dependent adherence decay and varying participation/compliance levels (30%–80%). Although reduced adherence increased ICERs for high-frequency strategies, the relative ranking and overall cost-effectiveness conclusions remained robust. Thus, while the base-case assumption may be optimistic for intensive screening, our findings are supported by scenario testing that accounts for more realistic adherence patterns.

Several limitations should also be acknowledged. First, while model parameters were carefully calibrated and validated to match real-world data, any mathematical model inherently involves simplifying assumptions and may affect outcome precision. Participation was 58.29% in the program, which may introduce selection bias; however, sensitivity analyses across varying adherence levels showed consistent cost-effectiveness results. Second, our model focuses on the conventional adenoma–carcinoma sequence, which accounts for over 95% of CRC cases in Asian populations^[24,25]^. SSLs and traditional serrated adenomas, which comprise a smaller proportion of CRCs, were not explicitly modeled due to limited data on their prevalence, progression, and detection in China. Excluding these lesions may slightly underestimate missed lesions and CRC prevention, potentially introducing a modest optimistic bias in cost-effectiveness estimates. Future studies should incorporate serrated pathway parameters once reliable Asian-specific data become available. Third, the model assumed consistent compliance across screening frequencies, although higher frequencies may reduce compliance due to concerns about pain, sedation, and competing life demands. Finally, this study was conducted in Zhejiang Province, a region with relatively abundant colonoscopy resources and robust health care financing, which may limit generalizability to less developed settings. In provinces with constrained capacity or in other LMICs, early initiation or intensive screening strategies may be less feasible and less cost-effective. Our findings should therefore be interpreted as reflecting potential benefits under favorable health care infrastructure. Future research should incorporate region-specific colonoscopy capacity, health care investment, and compliance patterns to generate more tailored policy recommendations. In practice, screening cost structures, reimbursement mechanisms, and out-of-pocket expenses can substantially influence participation and cost-effectiveness. Moreover, constraints in health care resources, trained personnel, and colonoscopy availability may delay diagnosis and follow-up, undermining clinical outcomes. Patient health literacy, cultural attitudes, and transportation access also play important roles in adherence and realized benefits. Thus, while RF-FIT appears cost-effective and feasible in Zhejiang, its adaptation to other regions should be aligned with local health care infrastructure and population characteristics to optimize implementation.

## Conclusion

This study marks a significant milestone in CRC screening in China, demonstrating the feasibility, effectiveness, and cost-effectiveness of a province-wide risk-based RF-FIT strategy. To maximize health benefits and equity, policy implications include phased rollout in areas with sufficient colonoscopy capacity, prioritization of high-risk populations (e.g., men, individuals with family history, or adverse lifestyle factors), and targeted support for high-burden rural regions to reduce participation gaps. Leveraging integrated care networks, such as the Medical Community Health Alliance, can enable a coordinated “risk assessment–screening–referral” workflow. These findings provide empirically grounded guidance for risk-adapted, high-impact screening and offer a replicable model for regions with constrained resources, promoting a shift from broad coverage toward precise, efficient CRC prevention.

## Data Availability

All data relevant to the study are included in the article or uploaded as online Supplemental information.
